# Evaluation of Aging Effect on the Durability of Antibacterial Treatments Applied on Textile Materials for the Automotive Industry

**DOI:** 10.1021/acsomega.4c01272

**Published:** 2024-06-11

**Authors:** Matilde Arese, Ilaria Mania, Valentina Brunella, Vito Guido Lambertini, Roberta Gorra

**Affiliations:** † Department of Chemistry, University of Turin, Via Pietro Giuria 7, 10125 Turin, Italy; ‡ Fiat Research center SCPA (CRF), Stellantis, Corso Settembrini 40, 10135 Turin, Italy; § Department of Agricultural, Forest and Food Sciences, University of Turin, Largo Paolo Braccini 2, Grugliasco, 10095 Turin, Italy

## Abstract

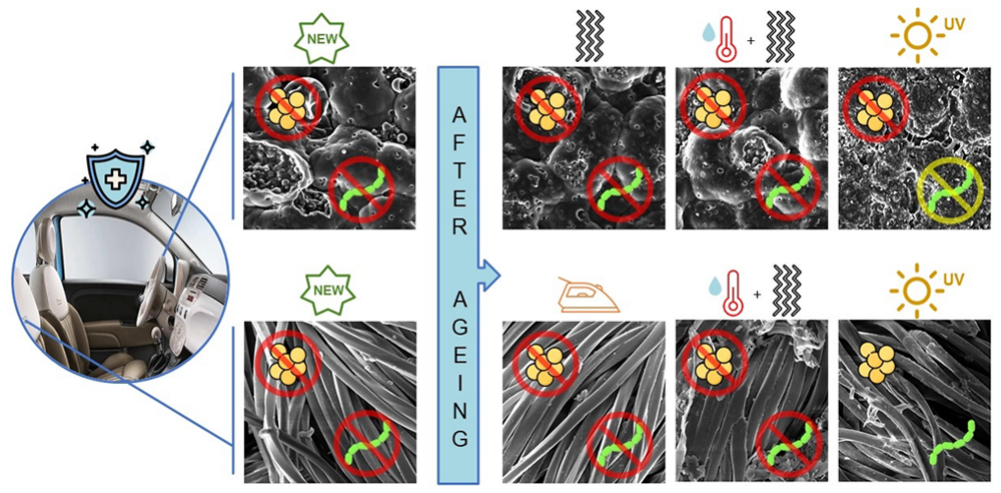

The automotive industry is always seeking novel solutions to improve the durability and the performance of textile materials used in vehicles. Indeed, especially after the coronavirus pandemic, antibacterial treatments have gained interest for their potential of ensuring cleanliness and safety toward microbial contamination within vehicles. This study gives a panoramic view of the durability of antibacterial treatments applied on textile materials in the automotive industry, focusing on their performance after experiencing accelerated aging processes. Two different textile materials, a fabric and a synthetic leather, both treated with antibacterial agents, were tested according to ISO 22196 and ISO 20743 standards, respectively, using two model microorganisms, *Escherichia coli* and *Staphylococcus aureus*. The impact of mechanical, thermal, and solar aging on the antibacterial properties has been evaluated. In addition, scanning electron microscope (SEM) analysis was performed to investigate the surface morphology of the materials before and after aging. Furthermore, contact angle measurements were conducted. The results suggest that neither mechanical nor thermal aging processes determined diminished antibacterial action. It was determined, instead, that the most damaging stressor for both textile materials was UV aging, causing severe surface alterations and a reduction in antibacterial activity.

## 1 Introduction

It is estimated that worldwide car sales grew to around 67.2 million automobiles in 2022 with a forecast to keep rising in 2023,^1^ considering that it is an undeniable fact that in today’s world a significant portion of our daily lives is spent in automobiles. This is particularly true for professions such as couriers, taxi drivers, and public bus operators, who spend their entire working day within the confines of a motor vehicle. Consequently, the environment inside motor vehicles has become a subject of increasing interest over recent times.^2^ In addition, during the past few years, the landscape of mobility has evolved significantly, ushering in a new paradigm that redefines urban life, movements, and transportation. Car-sharing services are developing at an ever-increasing level and have become a cornerstone of modern smart cities.^3,4^ This increasing phenomenon together with rising requests for higher hygiene standards, especially post-2020 due to the coronavirus pandemic, raised in the automotive industry the interest in textile finishing with antibacterial properties. Cars interiors are strictly close environments subjected to different conditions of heat and humidity,^5^ factors that have been shown to influence the growth rate and survival of many pathogenic microbes.^6−8^ Given that automotive interiors undergo minimal cleaning throughout their lifespan, ensuring hygiene in these settings is a topic of great concern. In a car, the number of bacteria can be huge, especially on parts exposed to frequent human interaction such as the steering wheel and gear stick, the seats, and even more the carpets.^9^ Moreover, when shared cars are considered, it becomes evident that a considerable number of individuals came into contact with these surfaces on a daily basis, facilitating the circulation of bacteria. Antimicrobial textiles nowadays are often used in hygienically demanding areas such as the food sector, hospitals, or nursing homes to improve the environmental hygiene and prevent nosocomial infections. They are also employed in sportswear fashion and in the military sector to prevent bacterial proliferation and infections and for odor control.^10,11^ It has been demonstrated that microorganisms can be transferred from contaminated textiles to surfaces, where they can persist for months, forming biofilms and leading to bacteria transmission.^12−14^

Antimicrobial effects in textiles can be achieved through the incorporation of a biocide molecule in the coating mixture applied during the finishing stage, by immersion in a solution containing the active antibacterial principle, also called “in foulard” treatment,^15^ or through the incorporation into fibers during the spinning process.^16^ Antimicrobial textiles can selectively target one type of microbe, e.g., bacteria, fungi, or viruses, or they can exhibit a broad-spectrum effects acting as antibacterial, antifungal, and antiviral agents simultaneously.^17^ The antimicrobial molecules can act as inhibitors of microbe growth or also kill them when they come in contact with the surface; for example, quaternary ammonium compounds interact electrostatically with bacterial surfaces, with their polar tails penetrating bacterial cell membranes, causing loss of cell integrity and consequently bacteria death.^18^ Another category of antimicrobials is constituted by metallic salts such as silver (Ag) salts, which act on the cellular metabolism inhibiting cell growth and proliferation of fungi and bacteria.^19,20^ Although there have been several studies and advances in the development of more effective antibacterial textiles in recent years, a key challenge remains to maintain antibacterial ability over time. Textiles with antibacterial properties applied in automotive interiors can be subjected to rubbing, cleaning operations, and conditions of heat and humidity which can cooperate to gradually diminish their antibacterial effectiveness after extended use.^21^ We considered two different types of textiles commonly used in the automotive industry^22^ to have a broad picture of the response of these materials toward several types of stress. The types of stress were chosen to better replicate the degradation condition panorama to which interior car trims are subjected during the vehicle lifespan, which corresponds, according to the study of Bonato et al., to around 15 years and 250 000 km;^23^ it has been reported in several studies, indeed, that mechanical,^24^ thermal,^25^ and UV aging^22^ can affect the performance of materials in car interiors.

To the best of our knowledge, this is one of the first studies on the durability of textiles for automotive application with antibacterial properties, where our aim was to reproduce the real stress textiles may be exposed to during their usage in a vehicle.

In order to give a broad picture of contrasting situations coexisting within a motor vehicle, two types of textiles, different in terms of material composition and antibacterial treatment, were chosen. The first was a synthetic leather applied to upholster steering wheels and gearshifts, and the second was a polyester fabric commonly used for seat covers.

Antibacterial activity, surface hydrophobicity, and surface morphology were tested before and after different stress treatments in order to assess the potential contribution of different stresses to the loss of antibacterial efficacy against *E. coli* and *S. aureus*, commonly used as model organisms in the International Organization for Standardization (ISO), particularly, we applied ISO 22196 for the synthetic leather and ISO 20743 for the fabric.

## 2 Materials and Methods

### 2.1 Textile Material and Experimental Setup

Two different types of automotive textile materials were selected. The first sample chosen is a poly(vinyl chloride) synthetic leather with a polyurethane finish. The biocide molecule was incorporated in the coating mixture applied on the aesthetic surface and integrated into several layers of the textile structure. The second is a polyester fabric with a polyurethane finish. The antibacterial properties were given through a process called “in foulard” treatment.^15^ This immersion ensured the thorough integration of the antibacterial components into the fabric’s matrix.

The synthetic leather was chosen, as it is the most used material for steering wheel application, together with real leather. As demonstrated in several studies, the steering wheel is one of the most contaminated components in a car interior,^26−28^ as it is subjected to direct contact with hand palm. Another part of a vehicle interior in contact with users for a long period is the seat, which is usually covered by synthetic fabric material such as polyester. Considering the porous nature of the material and the frequent and repeated contact with human skin, sweat, and contaminated clothes, also seats can be significant sources for the spread of microorganisms.^29^

In this article, we will not give information about the antibacterial treatment molecules and the effect toward bacterial strains because it was stipulated in an agreement with both materials’ suppliers which limits the information that can be provided regarding brand names and the specific antibacterial formulations.

The experimental setup that we followed for both synthetic leather and fabric textile samples consisted of first aging and stressing the material and then evaluating the antibacterial efficacy at time zero (t0) and after 24 h of incubation (t24).

For simplifying the presentation of this article, we assigned to each sample a different code: NTNt0 (sample without antibacterial treatment, not stressed, after 0 h of incubation), TNt0 (sample with antibacterial treatment, not stressed, after 0 h of incubation), NTNt24 (sample without antibacterial treatment, not stressed, after 24 h of incubation), TNt24 (sample with antibacterial treatment, not stressed, after 24 h of incubation), TWt24 (sample with antibacterial treatment, subjected to wear testing, after 24 h of incubation), TTWt24 (sample with antibacterial treatment, subjected to first thermal stress, simulated by a thermo-humid static chamber (CTUS), followed by wear testing, after 24 h of incubation), TSt24 (sample with antibacterial treatment, subjected to steaming test, after 24 h of incubation), and TLt24 (sample with antibacterial treatment, subjected to solar aging, after 24 h of incubation). In Table 1 are reported the samples tested and the type of aging to which they were subjected. Both synthetic leather and fabric were stressed through solar aging and a combination of wear testing and thermal stress, simulated by a thermo-humid static chamber. The single wear test was evaluated only for the synthetic leather, and the steaming was executed only on the fabric. This diversity is due to the different materials and nature of samples as well as the dissimilar application on the vehicle.

**Table 1:** Tested Samples and Type of Aging

sample	wear test	wear test + CTUS	steaming	solar aging
synthetic leather	yes	yes	no	yes
fabric	no	yes	yes	yes

### 2.2 Aging Instruments

Samples were subjected to three main forms of environmental stress: mechanical, thermal, and solar stress.

#### 2.2.1 Mechanical Stress

The mechanical stress was simulated through wear testing performed by an abrasimeter (Cesconi instrument).^24^ The purpose of this test is to simulate the rubbing of the clothes on the seat and the friction of the hands on the steering wheel. On both synthetic leather and fabric textiles, samples were treated with six thousand revolution movements through standard abrading fabric with an applied load of 3 kg.

#### 2.2.2 Thermal Stress

The thermal stress was simulated by the thermo-humid static chamber (CTUS), brand BAVA, used to recreate conditions of damp heat. Both samples were subjected to 40 °C and 90% relative humidity, without condensation, for 250 h.

Another type of thermal aging is the steaming test, which was executed only on the fabric. It simulates the process to eliminate wrinkles on the seat after the upholstering of the fabric on the seat’s foam. To replicate this action, the fabric was ironed nonstop for 3 s in all directions with considerable load with an iron protected by a Teflon shell.

#### 2.2.3 Solar Aging

To reproduce the solar aging caused by the UV rays, the Q-SUN Xe-2 Xenon Test Chamber, brand Q-Lab, was used. The irradiation source is an arc-xenon lamp with a quartz-boron filter, the specified radiant exposure is 0.55 W m^–2^ nm^–1^ at 340 nm. Considering the different final applications of the textile samples on the vehicle, they were subjected to distinct radiant exposure: the synthetic leather, used for steering wheel application, was subjected to 601 kJ/m^2^, while the fabric, used for seat application, was subjected to 225 kJ/m^2^.

### 2.3 Antibacterial Activity

The antibacterial activity on samples, new and after stress, was assessed by applying ISO 22196:2011 for the synthetic leather and ISO 20743:2021 for the fabric. ISO 22196 has been chosen as the antibacterial test method because is the most widely used standard procedure in the industry,^30,31^ which delineates an in vitro approach for evaluating antibacterial activity on treated plastics and other nonporous surfaces. For the antibacterial assessment on the fabric, ISO 20734, which is a frequently applied procedure for textile porous materials, has been selected.^32,14^ Samples of the same material not stressed and without antibacterial treatment were included in the test panel to allow the calculation of the antibacterial action. Some minor modifications were applied to the ISO methods and are reported in detail in Supporting Information. Briefly, both ISO methods involved the inoculation of a known aliquot of two model microorganisms, *Escherichia coli* ATCC 35150 and *Staphylococcus aureus* ATCC 25923, which are also common microorganisms that can be found in the car environment.^9,28^ The count evaluation in terms of colony forming units (CFU) was done immediately after the inoculation (sample t0) and after 24 h of incubation (sample t24).

According to the ISO standards, the antibacterial activity was expressed in percentage of bacterial reduction (*R*%), calculated as follows:
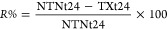
where NTNt24 is the bacterial load in CFU/cm^2^ for the sample without antibacterial treatment, not stressed, after 24 h of incubation and TXt24 is the bacterial load in CFU/cm^2^ for the sample with antibacterial treatment after 24 h of incubation where X = N indicates not stressed, X = W indicates the sample after wear testing, X = TW indicates samples after thermo-humid static chamber and wear testing, X = L indicates samples after the solar aging test, and X = S indicates samples after the steaming test (performed only on the fabric).

The presence of significant differences among bacterial counts obtained from samples undergoing different treatments was evaluated with one-way ANOVA, when the normal distribution of residuals and the homogeneity of variance criteria were met. Otherwise, the Kruskal–Wallis test was applied.

### 2.4 SEM

SEM analysis was performed with an Evo50 Zeiss SEM equipped with an energy-dispersive X-ray (EDX) detector. Morphological investigation was performed by scanning electron microscopy (SEM) with 15 kV scanning voltages and secondary electron detection. Before SEM analysis, both samples were adhered onto a round metal stub and coated with a 3 nm gold layer by VAC COAT DSR1 sputter coater. The test was performed on samples not contaminated by bacteria.

### 2.5 Contact Angle

The water contact angle (CA) was measured with a 5 μL deionized water droplet at room temperature with an optical contact angle meter, DSA30 Drop Shape Analyzer Kruss. The contact angle values and the corresponding standard deviation reported are averages of 10 measurements made on different areas of the sample surface. The test was performed on samples not contaminated by bacteria.

## 3 Results

### 3.1 Antibacterial Activity

Tables 2 and 3 present the results for synthetic leather and fabric samples, respectively, and both bacterial strains after 0 h (t0) and 24 h of incubation (t24). The results of the one-way ANOVA test and Kruskal–Wallis test are reported in Tables 2 and 3 using the letters a, b, and c. For the synthetic leather sample, the one-way ANOVA test was applied, while for the fabric, because the variance homogeneity was not satisfied, the Kruskal–Wallis test was used.

**Table 2:** Synthetic Leather Bacterial Load and Antibacterial Activity^a^

	bacterial load (log CFU/cm^2^)		antibacterial activity (%)	
sample	*E. coli*	*S. aureus*	*E. coli*	*S. aureus*
NTNt0	4.07 ± 0.08^ab^	4.00 ± 0.02^a^		
TNt0	4.11 ± 0.01^ab^	4.09 ± 0.07^a^		
NTNt24	5.48 ± 0.11^a^	4.08 ± 0.17^a^		
TNt24	0.49 ± 0.84^c^	1.19 ± 0.09^b^	100	99.88
TWt24	0.56 ± 0.89^c^	1.14 ± 0.70^b^	99.99	99.78
TTWt24	2.30 ± 0.69^bc^	1.31 ± 0.65^b^	99.77	99.72
TLt24	3.64 ± 2.07^ab^	1.06 ± 1.26^b^	89.14	98.15

a The letters a, b, and c placed as superscripts after the bacterial load values indicate significant differences assessed by one-way ANOVA test.

**Table 3:** Fabric Bacterial Load and Antibacterial Activity^a^

	bacterial load (log CFU/cm^2^)		antibacterial activity (%)	
sample	*E. coli*	*S. aureus*	*E. coli*	*S. aureus*
NTNt0	4.36 ± 0.07^ab^	3.65 ± 0.10^ab^		
TNt0	0.00 ± 0.00^/^	0.00 ± 0.00^/^		
NTNt24	7.03 ± 0.12^a^	7.29 ± 0.09^a^		
TNt24	0.76 ± 0.62^b^	3.27 ± 0.69^ab^	100	99.98
TSt24	1.16 ± 0.54^ab^	1.64 ± 1.22^b^	100	100
TTWt24	1.40 ± 2.69^b^	2.03 ± 1.78^b^	100	99.97
TLt24	4.71 ± 2.19^ab^	3.67 ± 2.98^ab^	89.44	91.81

a The letters a, b, and c placed as superscripts after the bacterial load values indicate significant differences assessed by the Kruskal–Wallis test.

#### 3.1.1 Synthetic Leather

Immediately after the inoculation, the same CFU concentration was recovered from both treated (TNt0) and untreated (NTNt0) samples.

After 24 h for NTNt24, bacterial growth of 1 order of magnitude for both *E. coli* and *S. aureus* was registered, while TNt24 showed a strong bacterial decrease with respect to the initial inoculum.

Regarding stressed samples after 24 h of incubation, for TWt24 inoculated with *E. coli* a load similar to TNt24 was measured, and comparable results were also found for *S. aureus*, while TTWt24 showed a bacterial load slightly higher than that of TNt24 especially for *E. coli*. Despite this, it is still possible to confirm that a high antibacterial action occurred against both bacterial strains. This outcome confirms the strong antibacterial action even after mechanical stress and a combination of thermal and mechanical stress. Different behaviors were observed for samples TLt24: For *E. coli* a significantly higher load, consistent with a decrease in antibacterial action, was reported. For *S. aureus* instead the bacterial load remained comparable to samples TNt24, TWt24, and TTWt24, synonymous with a strong antibacterial action.

#### 3.1.2 Fabric

The starting inoculum concentration of *E. coli* and *S. aureus* was quantified evaluating only the bacterial load of NTNt0 because, unlike the synthetic leather, on sample TNt0, no CFU were detected. In this case, the load gap between NTNt0 and NTNt24 was up to 4 orders of magnitude, while samples TNt24 gave excellent antibacterial action against both *E. coli* and *S. aureus*.

Regarding specimens after aging, for TWt24 and TTWt24 inoculated with *E. coli*, a load similar to TNt24 was found. Comparable results, even if more numerically variable (see Supporting Information), were also found for *S. aureus*, confirming the persistence of excellent antibacterial action even after mechanical stress and a combination of thermal and mechanical stress.

The solar aging determined, instead, an important decrease of the antibacterial action, with specimens TLt24 showing comparable load to NTNt24 samples for both *E. coli* and *S. aureus*.

### 3.2 SEM

**Figure 1:**
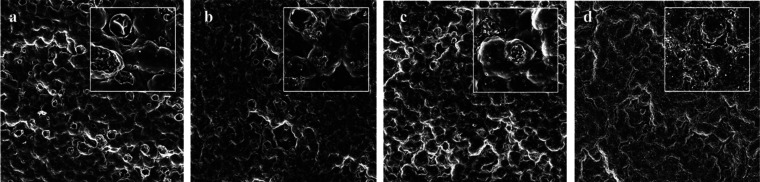
SEM images of synthetic leather samples TN (a), TW (b), TTW (c), and TL (d) with magnification ×500 (big square) and ×1400 (small square).

**Figure 2:**
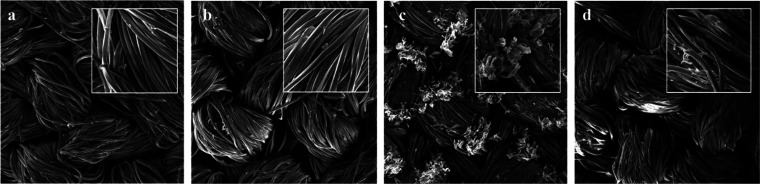
SEM images of fabric samples TN (a), TS (b), TTW (c), and TL (d) with magnification ×500 (big square) and ×1400 (small square).

Scanning electron microscopy was employed to collect data about the surfaces of both synthetic leather and fabric before and after subjecting them to stress. SEM images are presented in Figures 1 and Figure 2, with magnification ×500 (big square) and ×1400 (small square). The synthetic leather surface structure (Figure 1) was characterized by the presence of ridges and valleys, a typical pattern imprinted during the embossing process. Surfaces of samples TW (Figure 1b) and TTW (Figure 1c) did not show any differences compared to that of TN (Figure 1a). However, noteworthy modifications were identified in sample TL (Figure 1d): the surface appears damaged, and the ridges and valley patterns were less distinguishable compared to samples TN, TW, and TTW. Additionally, the size of the surface valleys was also measured. For samples TN, TW, and TTW the largest holes measured approximately 220 μm, while the smallest were around 160 μm. In contrast, for the sample TL, the overall measurements were around 120 μm. The images related to fabric samples (Figure 2) reveal single fibers pulled and twisted together to form the yarn. This intertwined structure is clearly visible in the 500× magnification images. Sample TS (Figure 2b) appears in both magnification stages remarkably similar to TN (Figure 2a) while some differences were noted in samples TTW (Figure 2c) and TL (Figure 2d). In the case of sample TTW (Figure 2c), the abrasimeter action caused flattening and breakage of the fibers. Instead, in sample TL, especially at magnification ×1400, irregularities in the shape of fibers were observed, indicating damage and brittleness. In this context, the gaps between the yarns were measured, and in all samples, they averaged around 320–360 μm.

### 3.3 Contact Angle

The results of the water contact angle analysis are shown in Table 4. For the synthetic leather all four samples show typical hydrophobic behavior,^33,34^ although TW and TTW have a lower contact angle value compared to TN and TL. For the fabric, the water droplet quickly spread and wet the fabric in all samples, indicating hydrophilic action.

**Table 4:** Contact Angle

synthetic leather		fabric	
sample	contact angle θ (deg)	sample	contact angle θ (deg)
TN	110.1 ± 5.01	TN	0
TW	89.1 ± 3.34	TS	0
TTW	89 ± 6.09	TTW	0
TL	104.2 ± 6.67	TL	0

## 4 Discussion

In this study, we assessed the potential effect of different stress factors on antimicrobial treatments applied on two textile materials in order to understand which type of induced degradation most affects the antibacterial treatment efficacy and how the morphology and the hierarchical structure of the substrate can influence the durability of the antibacterial properties after the mentioned stresses.

The first consideration emerging from this study is that different textile materials employed within vehicles support microbial growth in different ways. This is particularly clear when observing the untreated samples: while untreated synthetic leather allows only limited bacterial proliferation within 24 h (around 1 order of magnitude), untreated fabric displays an increase in bacterial load of more than 3 orders of magnitude for both the tested bacterial strains. This can be linked to the differences in terms of properties and morphology existing between the two materials. The fabric surface permeability and hydrophilicity may create an ideal environment for microbial growth by offering physical protection to the cells and facilitating moisture retention, as previously described for other textiles.^35^ Conversely, in synthetic leather, the surface hydrophobicity, assessed by contact angle results, determines a reduced bacterial adhesion on the surface due to the decrease in interaction and contact area. Increased hydrophobicity has indeed been recognized as a strategy to control bacterial growth by limiting the cell adhesion to surfaces.

Prakash et al. were able to reduce the bacterial growth on a Ti_6_Al_4_V surface by a patterned texture with micro/nanocraters which increase the hydrophobicity.^36^ Comparable results were obtained by Wang et. al, who developed a superhydrophobic surface-based gripper (SSBG) exhibiting antimicrobial properties thank to super-hydrophobicity which contributes to repeling bacterial adhesion on the surface.^37^ These observations suggest that, when studying the application of antimicrobial materials in the automotive sector, some materials and thus some vehicle components are more prone to microbial colonization and may take increased advantage in the application of an antimicrobial treatment.

Considering now the effects of the stresses on the tested materials, starting with the synthetic leather, mechanical stress and thermal stress, even if combined, did not decrease in a relevant way the antibacterial action against *E. coli* and *S. aureus*. Despite the diminution of the surface hydrophobicity, ascribable to the partial polyurethane coating damage caused by the mechanical abrasion of the wear test,^38,39^ the antibacterial action is preserved, probably thanks to the layer-by-layer antibacterial treatment application. In addition, if we consider SEM results, it is possible to notice that samples TW and TTW do not present any differences compared with sample TN, indicating the preservation of the surface morphology. However, UV aging led to a noticeable decrease in antibacterial efficacy against *E. coli*, while, under the same stress conditions, the antibacterial efficacy did not diminish toward *S. aureus*. The different sensitivity to bacterial proliferation experienced toward the two species can be interpreted as an effect of a distinct interaction of the bacteria with the surface. We excluded, indeed, damage to the antibacterial molecule, because if the antibacterial treatment were compromised, we would have expected a significant decrease in antibacterial activity against both bacterial strains.

The results of the SEM analysis can support this thesis. Observing sample TL, it is possible to see clearly the surface damage, including the formation of holes that can be attributed to photodegradation processes induced by UV radiation.^40^ It is demonstrated that the accelerated light exposure determines the formation of microvoids and microcracks in the PVC due to the relaxation of residual energy of the system, derived from the effects of dehydrochlorination, creation of polar groups, and the adjustment of conformation of macromolecular chains.^41^ Considering this, we assumed that *E. coli* was able to insert in the new cavities generated by the action of UV rays and proliferate more effectively, whereas *S. aureus* did not exhibit the same capability. Indeed, an important difference between *E. coli* and *S. aureus* is the shape morphology: *E. coli* bacterial cells have a rod-shaped morphology whereas *S. aureus* bacterial cells have a cocci-shaped morphology and are often present as grape-like clusters.^42^ In addition, Gram-negative and Gram-positive bacteria differ in terms of cell wall composition. Gram-negative bacteria comprise a cytoplasm surrounded by three layers made up of an inner surface/membrane, a layer of peptidoglycan, and an outer membrane; Gram-positive bacteria, in contrast, lack this outer membrane but possess a cytoplasmic membrane surrounded by a thick layer of peptidoglycan complemented by anionic glycopolymers known as teichoic acids.^43^ This is not surprising, since contrasting responses in the adhesion and proliferation of different model microorganisms to the same material undergoing surface topography or roughness alterations have been previously reported.^44−46^ However, understanding the mechanisms behind this differential behavior would require a deeper characterization of the studied surfaces, since it has been shown that, on the same material, bacterial response to surface morphology can vary in relation to other properties, such as surface chemistry.^47^ These differences may indeed justify the distinct bacteria proliferation observed.

Regarding the fabric, mechanical stress and thermal stress, even if combined, had minimal impact on antibacterial action against both *E. coli* and *S. aureus*. Analyzing SEM results, the surface morphology of sample TS does not present any differences compared to sample TN, while the action of the wear test caused damage to the yarns on sample TTW, but this did not significantly impact antibacterial efficacy. Considering instead the UV aging, it determined a stronger effect against both bacterial strains, with a slightly more significant reduction toward *S. aureus*. This result can be explained as a consequence of the photochemical degradation of both polyester fibers and antibacterial active molecules caused by the action of the UV aging process. Observing the SEM picture of sample TL, the yarns appeared brittle and damaged as a consequence of UV exposure. This result was also found by Pinlova and Nowack in a study investigating the same fabric material, which showed clear signs of structural damage with many fibers being broken off due to UV aging.^48^ Indeed, it was demonstrated in previous studies that UV weathering of textiles can lead to photochemical degradation of textiles fibers,^49,50^ which has been proposed to occur via chain scission leading to the generation of carboxyl end groups followed by the formation of mono- and dihydroxy terephthalates and aldehydes.^51,52^

## 5 Conclusion

The results of this study show how two of the materials most commonly found in car interiors, synthetic leather and polyester fabric, present intrinsic characteristics that, even in the absence of antimicrobial treatments, influenced their interactions with bacteria. Synthetic leather, being nonporous and hydrophobic, led to bacterial retention on the surface, while the fabric, with its permeable and hydrophilic nature, facilitated bacterial penetration and promoted increased bacterial proliferation. This suggests an increased need for the application of strategies for bacterial control for fibrous and porous textiles.

The tested materials showed good preservation of their antibacterial properties in response to mechanical and thermal stresses, but not to solar aging. However, the effect changed in relation to material and bacterial strain, leading to a generalized reduction in antibacterial efficacy of synthetic leather and to reduction of only activity against *E. coli* on the fabric. These results can have some practical implications for the automotive textile materials industry. First, they point out that solar aging should receive particular attention when testing the durability of materials with antibacterial properties applied to the automotive sector. Moreover, they highlight the importance of understanding the mechanisms behind the loss of efficacy, in order to predict the response of different microorganisms and develop new strategies for bacterial control to fulfill a durable and effective antibacterial effect.

Looking ahead, the integration of antibacterial fabrics could become a common standard not only in vehicles but also in other contexts such as public transportation, offices, and public spaces. This not only would improve public health but also could help reduce the costs associated with managing infections and communicable diseases.

## Supporting Information Available

Additional experimental details, materials, and methods, box plots of bacterial counts on synthetic leather and on fabric (PDF)
