# Supplementary material for: Evaluation of Aging Effect on the Durability of Antibacterial Treatments Applied on Textile Materials for the Automotive Industry
Source: ACS Omega. 2024 Jun 11;9(25):27169–76. doi: 10.1021/acsomega.4c01272 (PMC11209923; doi:10.1021/acsomega.4c01272)
Supplement: Supplementary file 1 — ao4c01272_si_001.pdf [file ao4c01272_si_001.pdf]

Supporting information

# Evaluation of ageing effect on the durability of antibacterial treatments applied on textiles materials for automotive industry.

*Matilde Arese[ab], Ilaria Mania[c*], Valentina Brunella[a], Vito Guido Lambertini[b], Roberta Gorra[c]*

[a] Department of Chemistry, University of Turin, Via Pietro Giuria 7, 10125 Turin, Italy

[b] Fiat Research center SCPA (CRF), Stellantis, Corso Settembrini 40, 10135 Turin, Italy

[c] Department of Agricultural, Forest and Food Sciences, University of Turin, Largo Paolo Braccini 2, 10095 Grugliasco, Turin, Italy

* Email: ilaria.mania@unito.it

## *S1. Test procedure*

### *S1.1. Synthetic leather*

The procedure for antimicrobial testing of the synthetic leather samples is based on the ISO 22196:2011 standard for the measurement of antibacterial activity on plastics and other non-porous surfaces. The material was cut into a square shape with a size of 50 mm × 50 mm, only in the case of specimens after solar ageing test the size of the specimens was 50 mm x 30 mm. The difference in size was due to the structure of the Q-SUN Xe-2 Xenon Test Chamber masks through which samples are settled in the instrument.

No sterilization was done because the starting bacterial load on the surface was negligible (data not shown).

The inoculum was prepared by diluting one loop of the tested bacteria, grown on Nutrient Agar (OXOID) for 24 hours at 35°C, in Nutrient Broth/500 (OXOID).

Samples were placed into Petri dishes and inoculated with 400 µL of bacterial suspensions obtained by diluting the bacterial culture in Nutrient Broth/500 to a final concentration of 2,5-10 × $10^5$ cells/ml. The inoculum was then covered with transparency films Tartan brand 3M sized 40 mm × 40 mm and gently pressed down to help the inoculum spreading to the edges of the film.

Inoculated samples were incubated for 24 hours at 35°C and 90% of relative humidity. Bacterial suspension was recovered by washing the samples with a solution made of 10 ml of Tryptic Soy Broth (VWR CHEMICALS) added with 1g/l of lecithin (Bongiovanni s.r.l) and 7g/l of Tween 80 (SCDLP broth). The washing liquid was serially diluted in phosphate buffer (0.04 g/l di $KH_2PO_4$, 0.49 g/L NaCl, pH 6.8-7.2), plated by inclusion in Plate Count Agar (VWR CHEMICALS) and incubated at 35°C for 24 hours. In order to determine the recovery rate of bacteria, samples T0 were washed immediately after bacterial inoculation, serially diluted, plated and incubated. In case of different sample shapes, the transparency film dimension, and the amount of both inoculum and SCDLP were adjusted in proportion.

*S.1.2. Fabric*

The procedure for antimicrobial testing of fabric samples is based on the ISO 20173:2021 standard for the measurement of antibacterial activity of textile products. The material was cut into a square shape with a size of 32 x 32 mm. We had to modify the size of samples subjected to the abrasimeter because of lack of material, in this case samples were cut into a square shape with a size of 16 x 16 mm. Due to the presence of a basal bacterial load on non-inoculated material (data not shown), samples were sprayed with 70 v/v% of EtOH and UV treated for 10 minutes front and back. The inoculum was prepared diluting a bacterial culture in TSB and let it grow for 24 hours at 37°C. Samples were placed into small jars and inoculated with 400 μL of bacterial suspensions, obtained by diluting the bacterial culture with TSB/20 to a final concentration of 1-3*$10^5$ UFC/mL. Inoculated samples were incubated for 24 hours at 37°C. Bacterial suspension was recovered by adding 20 ml of SCDLP broth in the jar and executing 5 rounds of 5s at Vortex to help the inoculum resuspend. The washing liquid was then serially diluted, plated by inclusion in Tryptic Soy Agar (VWR CHEMICALS) and incubated at 37°C for 24 hours. To determine the recovery rate of bacteria, samples T0 were washed immediately after the bacterial inoculation, serially diluted, plated, and incubated.

In case of different sample shapes, the transparency film dimension, and the amount of both inoculum and SCDLP were adjusted in proportion.

## *S2. Box plot*

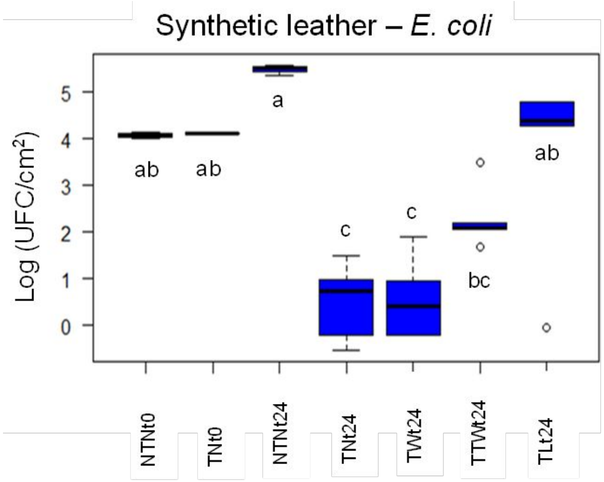


*Fig. S1: E. coli bacterial counts (after 0 and 24 hours of contact) of synthetic leather samples with and without antibacterial treatment. Different letters placed as superscripts on the bacterial load values indicate significant differences assessed by one-way ANOVA test.*

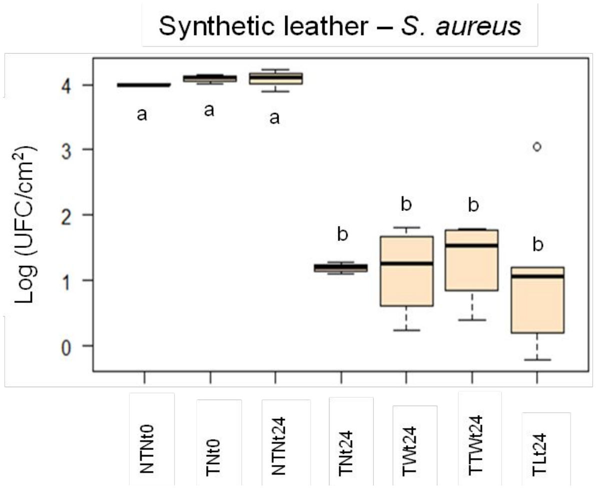


*Figure S2: S. aureus bacterial counts (after 0 and 24 hours of contact) of synthetic leather samples with and without antibacterial treatment. Different letters placed as superscripts on the bacterial load values indicate significant differences assessed by one-way ANOVA test.*

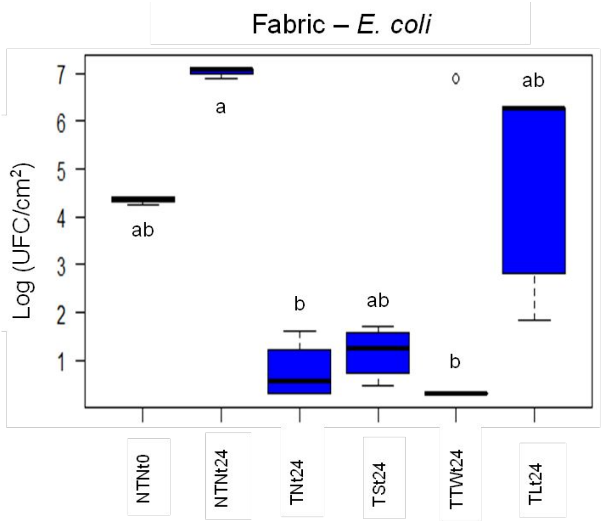


*Figure S3: E. coli bacterial counts (after 0 and 24 hours of contact) of fabric samples with and without antibacterial treatment. Different letters placed as superscripts on the bacterial load values indicate significant differences assessed by the Kruskal-Wallis test.*

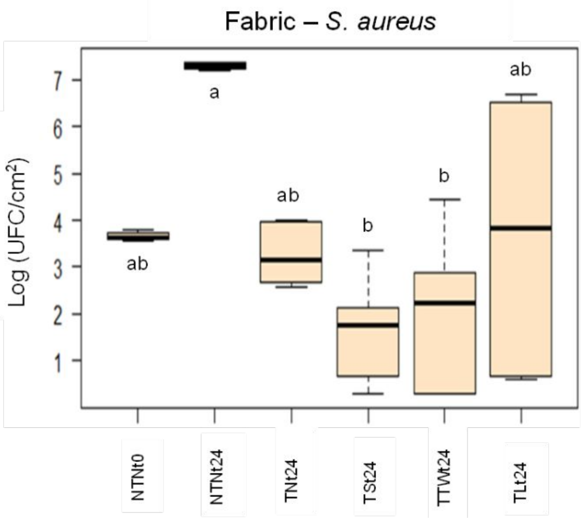


*Figure S4: S. aureus bacterial counts (after 0 and 24 hours of contact) of fabric samples with and without antibacterial treatment. Different letters placed as superscripts on the bacterial load values indicate significant differences assessed by the Kruskal-Wallis test.*
